# A Portable Gait Asymmetry Rehabilitation System for Individuals with Stroke Using a Vibrotactile Feedback

**DOI:** 10.1155/2015/375638

**Published:** 2015-06-16

**Authors:** Muhammad Raheel Afzal, Min-Kyun Oh, Chang-Hee Lee, Young Sook Park, Jungwon Yoon

**Affiliations:** ^1^School of Mechanical & Aerospace Engineering & ReCAPT, Gyeongsang National University, Jinju 660701, Republic of Korea; ^2^Department of Rehabilitation Medicine, Gyeongsang National University Hospital, Jinju 660702, Republic of Korea; ^3^Departments of Physical Medicine and Rehabilitation, Samsung Changwon Hospital, Sungkyunkwan University School of Medicine, Changwon 630723, Republic of Korea

## Abstract

Gait asymmetry caused by hemiparesis results in reduced gait efficiency and reduced activity levels. In this paper, a portable rehabilitation device is proposed that can serve as a tool in diagnosing gait abnormalities in individuals with stroke and has the capability of providing vibration feedback to help compensate for the asymmetric gait. Force-sensitive resistor (FSR) based insoles are used to detect ground contact and estimate stance time. A controller (Arduino) provides different vibration feedback based on the gait phase measurement. It also allows wireless interaction with a personal computer (PC) workstation using the XBee transceiver module, featuring data logging capabilities for subsequent analysis. Walking trials conducted with healthy young subjects allowed us to observe that the system can influence abnormality in the gait. The results of trials showed that a vibration cue based on temporal information was more effective than intensity information. With clinical experiments conducted for individuals with stroke, significant improvement in gait symmetry was observed with minimal disturbance caused to the balance and gait speed as an effect of the biofeedback. Future studies of the long-term rehabilitation effects of the proposed system and further improvements to the system will result in an inexpensive, easy-to-use, and effective rehabilitation device.

## 1. Introduction

Gait disturbance results in functional disability after stroke, and improving walking functionality is the most often stated goal of such subjects among all stroke-related impairments [1–5]. One-sided weakness, also known as hemiparesis, is the most common impairment pattern among the subjects suffering stroke, which can result in gait asymmetry. As gait symmetry and energy expenditure are related, the most efficient pattern is a symmetrical gait [6, 7]. Individuals with stroke have higher energy expenditures during gait and extremely low ambulatory activity levels compared with healthy controls [8, 9]. The poststroke asymmetric gait pattern appears to be an important factor in the increased energy expenditure observed. The musculoskeletal health of the nonparetic limb is also negatively affected by an asymmetric poststroke gait. High forces repeatedly applied to a limb can lead to pain and joint degeneration [10, 11]. In a poststroke gait, increased vertical ground reaction forces through the nonparetic lower limb are positively correlated with temporal asymmetry [12]. Thus, over time, the asymmetric gait of such subjects exposes the nonparetic limb to increased loading.

Several methods can be used to evaluate and diagnose gait problems, with different classifications according to the severity of the disorder, based on the level of functionality, compared with a healthy gait [13]. Clinical therapists apply specialized rehabilitative techniques to correct the abnormalities [13, 14]. The objective of gait rehabilitation is to increase the functional walking ability of the patient to an acceptable level for performing normal tasks, thus reducing risks for subsequent health defects. Due to variation in the causes and symptoms of gait disorders, rehabilitative methods are often focused on the individual patient [15]. Adequate gait rehabilitation demands committed time with a therapist, expensive instrumentation and training devices, and the use of a gait laboratory [15–17]. In stationary settings, force plates and force mats are used for their accuracy, but size, cost, and usability limit their implementation outside the clinic [18, 19]. Instrumented treadmills are able to gather large amounts of step data, but are limited by the controlled environment and prescribed walking pattern necessary [20]. Portable systems are also available to measure gait parameters [21–23]. Different implementations of these mobile systems have been evaluated and shown to provide accurate gait data, but they are expensive and complex equipment and the training required has limited their use [24–27].

Gait rehabilitation research has recently focused on the development of measurement and biofeedback devices. Novel air bladder sensors have been developed for gait analysis and have been used to provide visual feedback for correction of applied pressure under the foot [28, 29]. Visual-auditory feedback had been suggested for amputees, based on force sensors requiring a desktop computer for monitoring and feedback; the device is used in conjunction with a treadmill [30]. Recently, a force sensor insole and motion sensors were combined to record running data, demonstrating that the system is wearable with sensors for long periods of time and the data recorded by these sensors can be used for analysis of gait and movement [31]. The visual biofeedback devices implemented for individuals with stroke are either limited to use in providing assistance for improvement of quite standing, such as restoration of weight-shifting capacity and reduction in body sway [32, 33], or used with treadmill for gait assistance [34, 35]. Similarly the audio-biofeedback devices introduced to individuals suffering stroke can be used for assistance in quite standing and walking. Enhancement of weight-bearing has been reported to improve in poststroke using an audio-biofeedback device “SmartStep” [36]. Vibrotactile systems have been used recently in postural sway reduction and gait rehabilitation applications. Vibrotactile-based biofeedback systems were found to be an effective method in improving postural stability of healthy young subjects in complex standing tasks like quite stance on wobble board and tandem-Romberg stance [37, 38]. In community-dwelling elderly subjects, vibrotactile biofeedback delivered control of mediolateral sway during gait and reduction of fall risk [39]. Vibrotactile biofeedback reduced trunk sway during quite standing and locomotor activities in patients with vestibular loss [40–44]. Balance training in Parkinson's disease patients using a biofeedback system showed beneficial effects on trunk stability [45]. These studies demonstrate the effectiveness of vibrotactile biofeedback in providing instructional cueing. Vibrotactors can provide effective cues with the least interference to the subject's activities of daily life in portable biofeedback devices.

The purpose of this paper is to describe the design, manufacture, and verification of an inexpensive and portable gait rehabilitation biofeedback device for use by individuals with stroke. A major goal of this paper is to develop a system that can ultimately be available at low cost for use in gait symmetry rehabilitation outside of the traditional clinical environment and to demonstrate its effectiveness in enhancing gait symmetry using the proposed system. To our knowledge, this is the first reported system to apply vibration feedback to individuals with stroke for gait symmetry training. The current system is capable of determining common gait parameters through force-sensitive resistors (FSRs) embedded in a custom insole that can be easily implemented in patient's existing shoes. A waist-mounted microcontroller provides sensor sampling and vibrotactile biofeedback, as well as the ability to transmit real-time gait data wirelessly to a PC via an XBee module. By accurately measuring gait data and providing biofeedback to the user, the system provides an inexpensive and valuable tool for use in clinical rehabilitation. Using the proposed system, clinical trials were carried out in subjects suffering from poststroke gait asymmetry to evaluate and improve the effects of the device in reducing gait asymmetry. The effects of biofeedback were evaluated to provide efficient training with adequate feedback. Here, we describe our system, provide details of our subjects and protocols, and describe the parameters measured and our results. A discussion follows.

## 2. Materials and Methods

### 2.1. System Design

**Figure 1:**
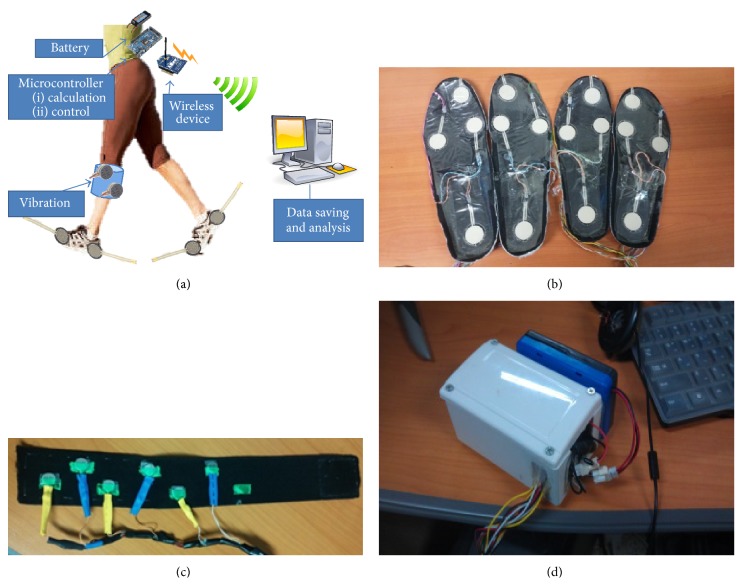
The device setup: (a) system diagram, (b) FSR-based insoles, (c) vibrotactors used for feedback, (d) controller with XBee transceiver and battery packed inside project case.

Individuals with stroke suffering from hemiparesis are highly motivated to recover a symmetrical gait cycle during normal walking. Temporal asymmetry measurement can be used to design a biofeedback system to assist patients in gait rehabilitation. To detect the temporal gait parameters, such as stance time and swing time, contact-based footswitches can be used. Change in pressure applied to a point can be detected using force-sensitive resistors (FSRs). A FSR-based footswitch system can work in the detection of foot contact with the ground, identifying the heel and toe contacts. This ground contact information for the right and left lower limbs is the basis of two important gait parameters: the time between first heel strike to toe off (stance time) and toe off to next heel strike (swing time). Manipulating these times can provide useful insight in determining gait abnormalities. The idea of our system is to match stance time for both legs and hence adopt temporal symmetry in the gait cycle, targeting subjects suffering from stroke. The wearable system is centered on an Arduino Due microcontroller board; the elements of the system are shown in Figure 1.

Our system collects heel and toe contact data with two insoles containing four FSRs on each insole, positioned at the heel, toe, fifth metatarsal, and first metatarsal. FSRs placed at the metatarsals allow validation in detecting ground contact. To ensure that male and female participants with diverse foot morphology could use this system, insole pairs of various sizes (Korean shoe sizes: 230, 240, 260, and 280) were built. A pair of insoles are connected physically to the Arduino board with wires; FSR signals are sampled at 1 kHz. The analog inputs of the Arduino board continuously read the FSR signals. The Arduino board calculates the stance time on each side by reading the FSR sensors and identifying ground contact. FSRs (A401 by Tekscan) utilized in insoles are capable of measuring forces up to 30 kN with a response time of less than 5 microseconds, linearity within ±3%, repeatability within ±2.5% of full scale, and hysteresis of less than 4% of full scale [46].

The Arduino board is connected to the XBee [47] transceiver module (Series 1 PRO) through the serial communication port, and the XBee can wirelessly send data to a personal computer (PC) or wirelessly receive a command signal (paretic/handicapped side, vibration mode, and target symmetry ratio) from the PC. The wireless transceiver module also allows monitoring of FSR sensor data for calibration purposes. The portable system is connected with a 2100 mAh polymer lithium-ion battery, allowing an ample continuous operation time of 8 h. A small project case is used to enclose all the circuitry; the case size is 9 × 12 × 5 cm and the whole system, including the battery, weighs about 450 g. An elastic belt, containing an array of six vibrotactors securely attached to the inside of the belt, is used for the vibrotactile biofeedback application on the subject. The vibrotactors used in our system are coin style, with a diameter of 10 mm and body length of 2.7 mm. Each vibrotactor operates at 3.3 V and 66 mA, producing an amplitude of 1.4 G [48]. The belt is worn by the subjects on the lower leg (between the knee and calf) instead of an in-shoe vibrotactor, so it should not interfere with proprioceptor information from the ground, as reduced feedback due to proprioceptive loss is likely to impair balance [49]. Also, direct muscle stimulation could contribute in enhancing the gait modification through afferent signal of vibration [50, 51]. The six vibrotactors cover the whole shank, from front to back. The vibrotactor array is interfaced with Arduino board's output to provide vibrotactile biofeedback. Control signals are proportionally generated on the Arduino board to drive vibrotactors. The modulation of intensity and duration is performed inside the Arduino, which generates the control signal proportionally depending on the mode of operation. An interface circuit provides capability of utilizing six vibrotactors simultaneously at 200 Hz and 3.3 V. The on/off status of the vibration signal can be visualized using the LED light connected to the array of vibrotactors. The whole system can be worn easily using elastic Velcro belts.

### 2.2. Vibration Modes

**Figure 2:**
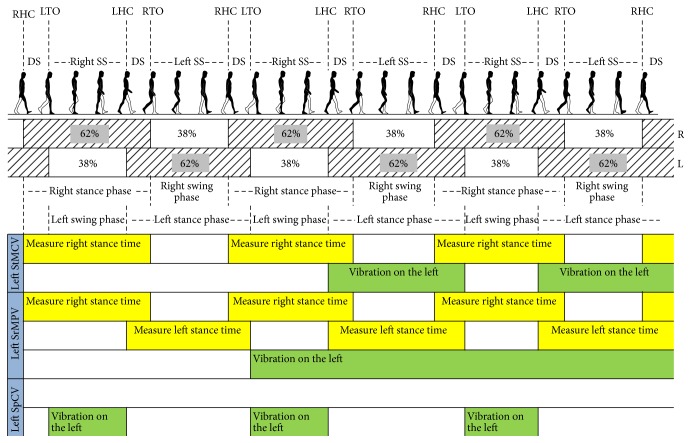
Modes of vibration explained with left-side supposed to be paretic/handicapped. (RHC: right heel contact, DS: double support, LTO: left toe off, Right SS: right single support, LHC: left heel contact, RTO: right toe off, and Left SS: left single support).

To achieve symmetry, subjects using this device get vibrational cues provided through the vibrotactors. Characteristics of the vibrational cue can differ to identify parameters of information being supplied. Currently, the system features three vibration modes: stance time matching constant vibration (StMCV) mode, symmetry ratio matching proportional vibration (SrMPV) mode, and swing phase constant vibration (SpCV) mode (Figure 2). StMCV and SrMPV modes provide cues targeting gait modification with information supplied in terms of vibration time and intensity, respectively. SpCV mode provides information on the swing phase to modify gait parameters.

In StMCV mode, vibration with constant intensity is provided on the handicapped/paretic side; vibration starts at heel strike and stays for time equal to the measured stance time of healthy side. In this mode, amplitude of vibration is 1.4 G and frequency of vibration is 200 Hz. In SrMPV mode, continuous vibration during the whole gait is provided on the handicapped/paretic side with varied vibration intensity. The vibration intensity is varied proportionally with difference between measured and target symmetry ratio. The target symmetry ratio is matched with the measured symmetry ratio of the previous cycle. If the measured symmetry ratio is close to the target symmetry ratio, the vibration intensity is less and vice versa. In this mode, amplitude of vibration is varied between 0 and 1.4 G and frequency of vibration remains same which is 200 Hz. In SpCV mode, vibration with constant intensity is provided during the swing phase, regardless of the symmetry ratio and stance time of the healthy leg. In this mode, amplitude of vibration is 1.4 G and similarly frequency of vibration is 200 Hz.

### 2.3. Study Participants

To use the system for gait rehabilitation in individuals with stroke, it was first necessary to check the credibility and functionality of the system. For this purpose, five healthy young subjects (age 26.2 ± 3.27 years, males, weight 72.3 ± 5.63 kg, height 170.8 ± 10.68 cm) were recruited to check the effectiveness of influencing gait parameters by our proposed system. The subjects had no history of musculoskeletal or neurological disorders. Trials with healthy young subjects were performed to show whether gait parameters measured with the proposed system could be used effectively to identify gait symmetry and provide biofeedback.

We also performed trials for proof-of-concept with four individuals with stroke to validate the use of our proposed system in increasing gait symmetry for patients and to demonstrate the possibility of implementing biofeedback in a gait training system. Demographic details of these subjects are provided in Table 1.

All individuals with stroke were inpatients of the Rehabilitation Center of Gyeongsang National University Hospital (Jinju, Republic of Korea). All subjects suffering stroke had clear symptoms of lower extremity weakness on the paretic side. All subjects gave written informed consent in accordance with the rules of our local Ethics Committee.

### 2.4. Experimental Protocol

**Figure 3:**
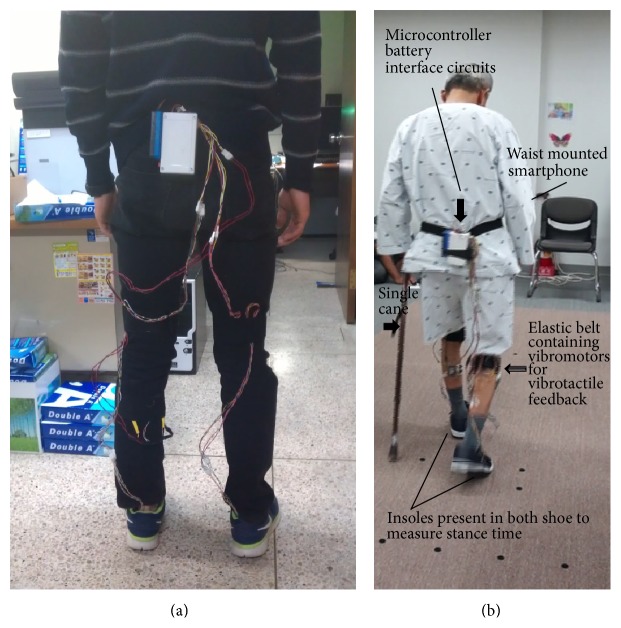
Device attachment: (a) healthy young subject and (b) individual with stroke.

Each subject was first introduced to the system and its use, and given instructions on how to interpret the feedback. Each subject installed the insole system inside their shoes and affixed the device to their waist over their clothes, using the easily attachable Velcro belts, which also handle the compact wire connections between the Arduino board and the insoles (Figure 3). Once the initial setup was complete, the subject was asked to walk in an empty, spacious area. FSR values acquired from the insoles on ground contact may vary depending on the participant, so prior to trials a preliminary walk was performed by each subject to determine the ground contact detection threshold. Each trial was conducted with different tasks given to the subject randomly. During each walking trial, the system is initialized from the PC via the XBee transceiver, which receives commands from the PC according to trial conditions and initializes the onboard processor. The system starts sending gait data to the PC and the PC logs the gait data for post-experimental analysis. All subjects performed trials at their self-preferred walking speed.

In our experimental trials different vibration modes were selected for the subjects (Table 2).

Healthy young subjects were asked to participate in walking tests to assess the system's ability to influence gait. The trials on healthy young subjects were conducted with seven different scenarios: normal walk, walk with left-side handicapped without vibration, walk with left-side handicapped with StMCV mode, walk with left-side handicapped with SrMPV mode, walk with right-side handicapped without vibration, walk with right-side handicapped with StMCV mode, and walk with right-side handicapped with SrMPV mode. In the normal walk scenario, the subjects were asked to walk with their natural gait and gait data were gathered. In the handicapped situation, the subjects were asked to induce the effect of gait asymmetry, so that the stance time of the handicapped side was double that of the healthy side (target symmetry ratio = 0.5). The aim of the trial was to demonstrate that a specific target symmetry ratio could be achieved by providing feedback. A 10 m randomized walking trial was set up for the healthy young subjects.

Individuals with stroke suffer from temporal asymmetry in their normal walk. Our experiments with such subjects were intended to demonstrate how the use of biofeedback could be helpful in improving temporal symmetry in gait. For these subjects, a 6 m walking trial was set up. Trials with subjects suffering from stroke were conducted in three different scenarios: normal walk, walk with StMCV mode, and walk with SpCV mode. Subjects suffering from stroke were more comfortable in walking trials with use of their ordinary walking aid (single cane); thus all subjects used their canes in all trials. To improve gait symmetry using the vibrotactors' cue provided, the patients were asked to match the stance time on both lower limbs (target symmetry ratio = 1.0) by interpreting the vibrational feedback.

### 2.5. Data Collection and Analysis

**Figure 4:**
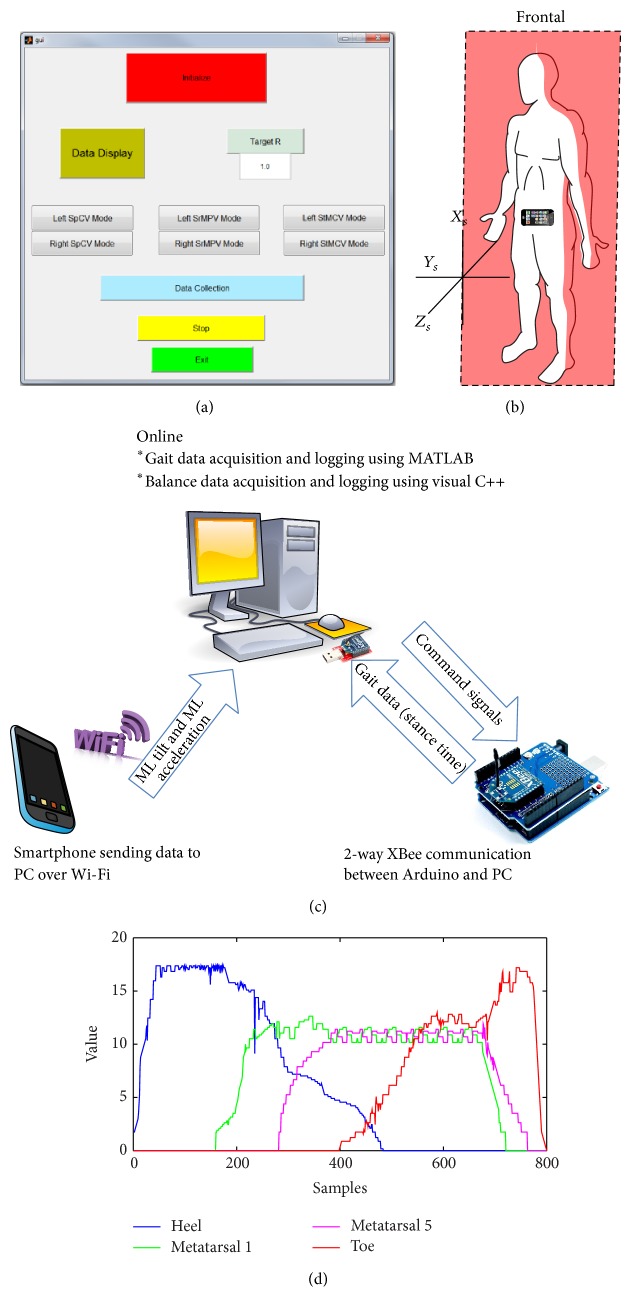
(a) MATLAB GUI, (b) smartphone placement, (c) communication network of the system, and (d) FSR data of one insole as read for calibration of the system.

All walking trials were conducted with coordination of the PC and an operator. A custom-built MATLAB GUI (Figure 4) was used to handle a two-way real-time communication with the Arduino board. The live link was set up between Arduino and MATLAB with the use of XBee transceivers ensuring real-time communication. The Arduino communicates with MATLAB at the baud rate of 57600 bps. Through the GUI, command signals (decimal format) were sent to Arduino board, which identify these command signals for determining the vibration mode, target symmetry ratio, and handicapped/paretic side (right or left) with a communication delay of approximately 10 ms. The gait data as a packet (sent from Arduino as a decimal format) was sent at the end of each gait cycle with the nearly same communication delay of 10 ms. Calibration of the system was performed by analyzing the sampled data of FSRs from an insole (Figure 4); utilizing this data, threshold for ground contact detection was determined. In each walking trial conducted with the subjects who suffered stroke, an Android smartphone was also used to identify mediolateral (ML) tilt and acceleration. The smart phone was physically attached to the waist with a leather belt. The frontal plane of the body was aligned with the *X*_*s*_ *Y*_*s*_ plane of the phone to allow measurement of ML tilt and acceleration (Figure 4). An Android application running continuously on the smartphone calculated and sent data (String format) over Wi-Fi. The data sent from the smartphone were received on a router physically connected to the particular PC used in the experiment. The PC was also running a custom-built software written in Visual C++. It monitored the network card continuously, decoded data packets from the smart phone, and stored data for post-experiment analysis. Recently, we used smartphone as a reliable tool to assess body sway parameters and designed a biofeedback system [52]. Now our custom-made android program can identify ML tilt and ML acceleration and provide this information to a socket program on the PC along with information of time at 100 Hz. Walking distance (6 m) was a known parameter and time spent during each walk was extracted from the smartphone data to calculate the gait speed during trials. For trials of subjects who suffered stroke, gait speed was calculated after experiments, to observe how it was affected under the use of our vibrotactile device.

The distance of walking trial was 10 m and 6 m for participants who were healthy young and who suffered stroke, respectively. For trials of both subject groups, we utilized a margin of 2 m, before and after the specified distances, to make sure of the exclusion of the gait initiation and termination steps. Each subject (healthy young and individual with stroke) performed each trial twice, an average of the measured gait data was utilized for analysis. The gait data logged for post-experimental analysis can be assessed in a more comprehensive and easily interpretable way by the use of symmetry ratio (*R*) [53], defined as

(1) $$\begin{array}{c} R=\frac{\text{Stance time of healthy side}}{\text{Stance time of Paretic or Handicapped side}}. \end{array}$$

The symmetry ratio (*R*) is considered for assessing gait modification/improvement. For individuals with stroke, ML tilt (RMS) and ML acceleration (RMS) were observed to determine the balance condition. To assess the significance of gait modification/improvement upon provision of feedback, a one-way ANOVA was used.

## 3. Results

### 3.1. Results of Healthy Young Subjects

**Figure 5:**
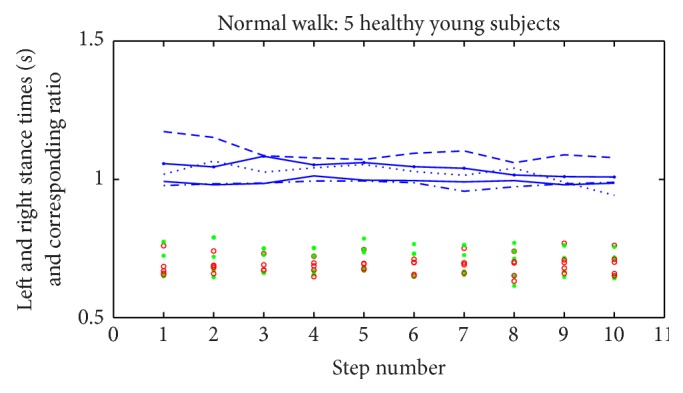
Normal walking trials of healthy young subjects. The stance time of the right side is marked by red circles and the stance time of the left side is marked by green asterisks. The distinct lines show the corresponding ratio (right to left) for each subject.

The vibrotactile cues provided intuitive feedback to the subjects; it helped in influencing gait symmetry without intensive training. In the normal walking test conducted with healthy young subjects, the ratio was calculated using right leg stance time over left leg stance time, which is expected to be unity as the subjects walked with their natural gaits. The results of this trial were as follows for the five subjects: 1.098 ± 0.02, 1.022 ± 0.03, 0.991 ± 0.01, 1.041 ± 0.02, and 0.983 ± 0.01. The data stored from this experiment are displayed in Figure 5.

**Figure 6:**
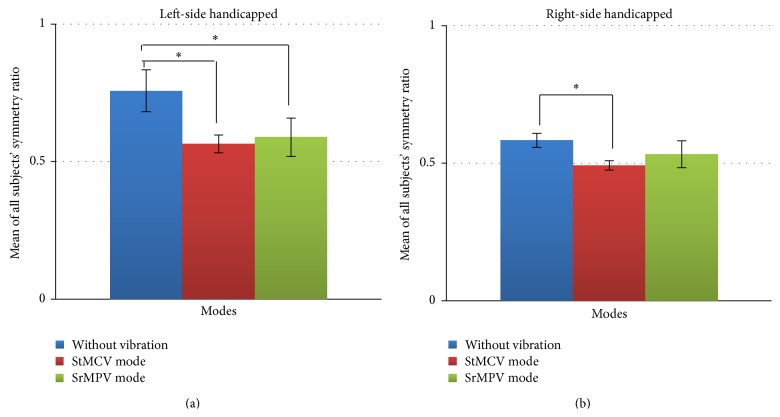
Comparison of vibration modes for trials of healthy young subjects in achieving target symmetry ratio (^*∗*^ *p* < 0.05).

Trials with healthy young subjects performing left-side handicapped walk and right-side handicapped walk were also conducted in three separate conditions for each: without vibration, StMCV mode, and SrMPV mode.  Table 3 shows the symmetry ratio (*R*) determined from the gait data of these trials.  Figure 6 shows a more comparative analysis of the effects of different vibration modes in achieving the target symmetry ratio. These values are the means and standard deviation of *R* collected over the 10 m walking trial.

Here, Exp. *R* is the expected symmetry ratio and S [1–5] is the subject number. Using ANOVA, we determined *p*-values for left handicapped without vibration versus StMCV mode (*p* = 0.0008), left handicapped without vibration versus SrMPV mode (*p* = 0.0065), right handicapped without vibration versus StMCV mode (*p* = 0.0002), and right handicapped without vibration versus SrMPV mode (*p* = 0.0823). In all five participants, lateral dominance resulted in reduced efforts and deviations in achieving target symmetry ratio during the right-side handicapped trials. The *p*-values indicate that feedback effectively provided cues to modify the gait of healthy young subjects in achieving the target symmetry ratios.

### 3.2. Results of Subjects Suffering from Stroke

Trials with subjects suffering from stroke were conducted in a similar manner, in which subjects were asked to walk in three scenarios: normal walk, walk with StMCV mode, and walk with SpCV mode. Patients used the vibration modes and attempted to improve their temporal symmetry in stance time. Tables 4 and 5 show the results for these subjects.

**Figure 7:**
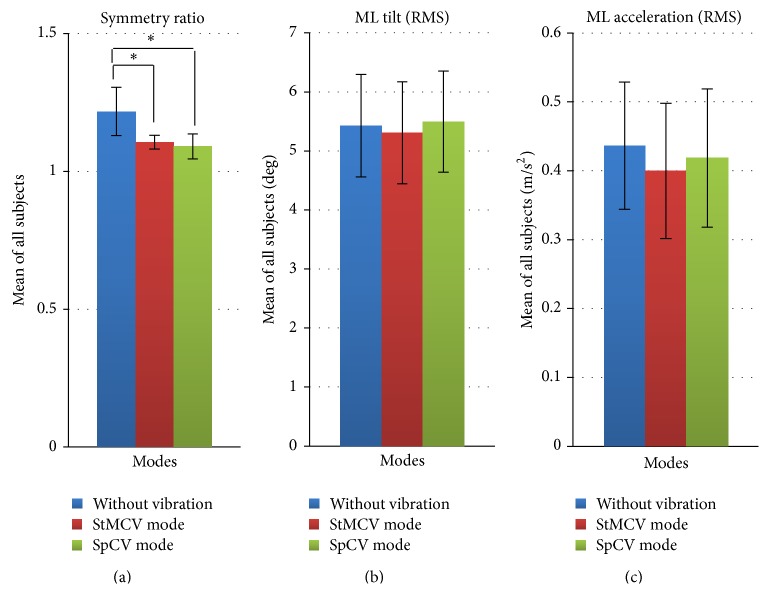
Comparative analysis of different vibration modes in trials conducted with subjects suffering from stroke (^*∗*^ *p* < 0.05).

In Table 4, the symmetry ratio (*R*) is the mean value collected over 6 m walking to identify the effect of the proposed system on gait symmetry. The ML tilt (RMS) and acceleration (RMS) shows the body balance measured during trials. Using ANOVA for symmetry ratios listed in the table, we determined *p*-values for without vibration versus StMCV mode (*p* = 0.0493) and without vibration versus SpCV mode (*p* = 0.0427). Using ANOVA for ML tilt gave *p*-values for without vibration versus StMCV mode (*p* = 0.8489) and without vibration versus SpCV mode (*p* = 0.9143). Similarly, *p*-values for ML acceleration were calculated using ANOVA for without vibration versus StMCV mode (*p* = 0.6077) and without vibration versus SpCV mode (*p* = 0.8006). The results are shown in Figure 7 and represent a comprehensive analysis of the trials conducted for individuals with stroke.

Comparative analysis of the right leg stance time, left leg stance time, and the gait speed, over 6 m walking is shown in Table 5. Improvement in stance time of the paretic side is evident with comparison of without vibration and vibration modes. Similar to balance of these subjects, gait speed was not disturbed on provision of vibrotactile feedback (without vibration versus StMCV mode *p* = 0.7810 and versus SpCV mode *p* = 0.8823). Increase in stance time on paretic side was noticeable (without vibration versus StMCV mode *p* = 0.1014 and versus SpCV mode *p* = 0.2106) but not significant enough to have *p*-value <0.05. Nonparetic side experienced slight changes in stance times but they were highly insignificant (without vibration versus StMCV mode *p* = 0.7913 and versus SpCV mode *p* = 0.4401).

**Figure 8:**
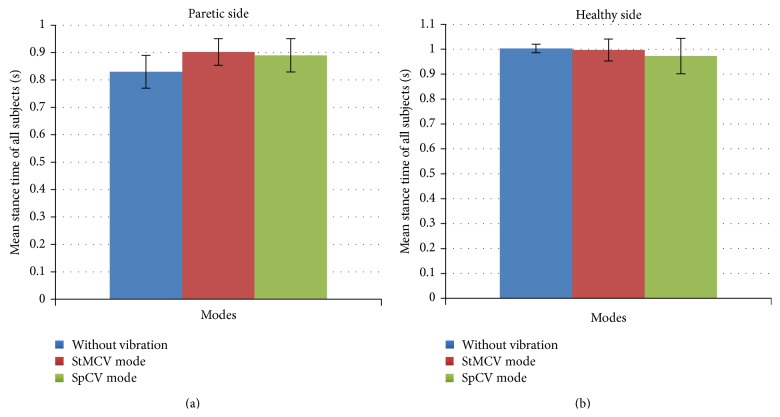
Comparative analysis of stance time on provision of different vibration modes.

Change in stance time on the provision of vibration modes is represented in Figure 8. On the paretic side provision of feedback caused an increase of stance time for both vibration modes. Mean increase in stance time of the paretic side was 72 milliseconds for the StMCV mode and 59 milliseconds for the SpCV mode.

## 4. Discussion

Stroke induces gait asymmetry as an after effect, resulting in decreased ambulatory activities and increased energy expenditure for mobility. The current methods for addressing a gait abnormality in the clinical setting are to establish a diagnosis and then prescribe a treatment [13]. Error-reduction and error-augmentation are two of the major control strategies employed for gait training robots [49]. Error-reduction is applied more often and it includes the impedance control and assist-as-needed paradigm. Impedance control may ensure correct kinematics but may impair motor learning effects. In the assist-as-needed paradigm gait training robot interferes in phases which cannot be performed independently by the patients. Error-augmentation based controllers make a function or movement more difficult than real task. This is based on the assumed importance of error correction in motor learning, with the hypothesis that error amplification leads to an increased rate of motor skill acquisition. Currently we employed error-reduction method and plan to consider the comparison of error-reduction and error-augmentation in the vibration feedback as a future work. A recent study demonstrated the possibility that poststroke asymmetric walking patterns could be remediated utilizing the split-belt treadmill as a long-term rehabilitation strategy [54]. Likewise, turning-based treadmill training may be a feasible and effective strategy to improve turning ability, gait symmetry, muscle strength, and balance control for individuals with chronic stroke [55]. These treadmill based rehabilitation strategies outcome promising results on the expense of high cost, complex protocols, and therapists' involvement throughout the training. Gait asymmetry usually occurs as a result of the difficulty in loading the paretic lower extremity during stance [56–58]. Thus, our system is based on the idea of matching stance time for both legs and adopting temporal symmetry in the gait cycle. Our system proposes a tool to gather gait data on the subject, as well as a subsequent treatment device. The inexpensive and compact system features the measurement of gait data in real-time, which can be further logged wirelessly on a PC for post-experimental analysis. Also, biofeedback based on the gait data, which can influence gait symmetry, can be provided by our system through vibrotactors. Human tactile perception is robust and suitable for multimodal sensing [59]. Our system influenced gait symmetry with a feedback signal provided as a vibration to the subject. They were able to use the cue and significantly modify gait without extensive training. This form of feedback could easily help the users during daily life activities. With an estimated prototype cost of US$ 300, our system provides an economical solution, compared with the more expensive ones currently available. Customizable feedback can be designed because the system is modular and easily modifiable.

### 4.1. Effects of Vibrotactile Feedback on Gait Symmetry

Experiments conducted on healthy young subjects supported the validity of the system. Trial results from normal walking by healthy young subjects confirmed that the system can measure gait data and useful information for providing biofeedback can be extracted from it. In the trials with a handicapped scenario, the healthy young subjects were asked to achieve a target symmetry ratio of 0.5; that is, the stance time of the handicapped side should be double the stance time of the healthy side. Without vibration signals, the subjects were unable to achieve the target in either left or right handicapped situations with large deviations from the achieved symmetry ratio. When using StMCV mode, the results were improved significantly, as indicated by the *p*-values (*p* = 0.0008, 0.0002) compared with the results of trials without feedback, with smaller deviation from the achieved symmetry ratio. Similarly, using SrMPV mode, healthy young subjects had smaller deviations from the achieved symmetry ratio and, compared with the no vibration mode, the target symmetry ratio was approached well, as indicated by the *p*-values (*p* = 0.0065, 0.0823). Provision of feedback contributed to the reduced deviation in achieving target symmetry ratio in all trials. Healthy young subjects acquainted well with the matching temporal information provided from StMCV mode in comparison to the proportional intensity information provided from SrMPV, which may be due to the difficulty of identification in variation of vibration intensity during walk. Time is objective and absolute, so it can be easily interpreted, whereas intensity is relative and depends on personal perception. Although both biofeedback modes improved the chances of achieving the target symmetry ratio, neither was actually able to comprehensively achieve an exact value. This inability to achieve a target symmetry ratio may be due to the lack of prior training on the system. From these results, it was decided to use StMCV mode for tests with subjects suffering from stroke. Before conducting trials on individuals with stroke, rehabilitation therapists in our team suggested use of SpCV mode and that it might provide another effective dimension of feedback.

The trials conducted with subjects suffering from stroke allowed the clinical assessment of the system and its effectiveness for rehabilitation. It was observed from the results that gait asymmetry existed even with the use of a walking aid (cane). During clinical trials, LED light allowed therapists to observe the vibration signals being generated visually. In two of the patient trials, toe contact was not detectable consistently due to an irregular walking pattern, so FSRs placed at the metatarsal were used to calculate the stance time. The biofeedback provision with the use of our system induced an effective influence on the gait symmetry as observed in the StMCV mode (*p* = 0.0493) although target ratio was not fully achieved since it requires extensive training programs and rehabilitation. Also, measured symmetry ratio indicates large deviations while utilizing the feedback to improve gait symmetry. This result correlates with the earlier findings from the LEAFS system [17] that large permanent gait changes must be made gradually. The SpCV mode provided a constant vibration during swing phase to reduce the spasticity and hence improving the smoothness of lower extremity during gait cycle. Trials conducting with SpCV mode also showed significant improvements in gait symmetry (*p* = 0.0427). This result may be due to broader role of proprioception and some central structures during human locomotion. Similarly, step-synchronized vibration stimulation of the soles improved gait steadiness in Parkinson's disease patients with predominantly balance impairment, presumably by enhancing sensory feedback [60]. Proprioceptive afferents can play a key role in calibrating the spatial motor frame of reference and provide a powerful sensory augmentation to the central nervous system [61]. The afferent signals due to vibration increase the excitability of several segments of the spinal cord and could facilitate triggering of locomotor-like movements [50, 51]. In a recent study it was found that auditory-motor synchronization was more stable during treadmill walking for double-metronome than single-metronome conditions, with subjects suffering from stroke exhibiting an overall weaker coupling of footfalls to metronome beats than controls [62], and this result could be due to the high cognition demands of audio-biofeedback in such system as the patients with least Mini Mental State Examination (MMSE) exhibited least adoption. A case study has been reported with two subjects suffering from stroke, providing combined visual and proprioceptive feedback employed with treadmill walking. 6-week training with the system resulted in improved gait speed and spatiotemporal symmetry [35]. Use of vibrotactile feedback for individuals with stroke in improvement of somatosensory function resulted in reduced body sway during quite stance and normal walk [63]. Our system provides a simplified vibrotactile cuing to modify gait. Our trials were conducted with no prior training to the system, yet significant improvement was observed in stance time symmetry during normal gait. After trials, individuals with stroke discussed the convenience of vibration modes and reported that SpCV mode was comfortable, which in their words “provided an effect of massage during swing phase of the walk and helped in reducing plasticity.”

In the post-experiment data analysis, Pearson correlation coefficients were calculated for ML tilt (RMS) and ML acceleration (RMS), with modes of without vibration, StMCV mode, and SpCV mode, and were found to be linear (Table 6).

These results clearly show that the balance of the body is not disturbed regardless of vibrational cues generated from the device. The cues were intuitive and helpful in reducing asymmetry, showing a high level of effectiveness in influencing the gait with no significant disturbance in the ML tilt (RMS) or ML acceleration (RMS), in conjunction with the results of Figures 7(b) and 7(c). The vibrational cues provided by the system helped the individuals with stroke in loading the paretic lower extremity during stance (Table 5), contributing effectively to reducing gait symmetry (Table 4). Moreover, balance and gait speed were not disturbed by provision of the vibrotactile feedback.

Biofeedback method selection is an imperative task; various researches have shown the pros and cons of utilizing the specific feedback method in poststroke rehabilitation. In our system, StMCV mode provided a vibrational cue of time and challenged subjects to follow that time of vibration to match with their stance time of handicapped/paretic side. Lee et al. reported a similar approach of attractive cuing [64], where healthy young subjects successfully replicated the task of slowly bending at waist using attractive vibrotactile instructional cues. Likewise in our system, SrMPV mode provided a vibrational cue of varied intensity, inspiring the users to reduce the vibrations until they could match their symmetry ratio with target. Such a method for achieving target symmetry ratio showed promising results in a recent research of gait feedback and training [65], where healthy young subjects were provided with vibrotactile feedback on a smartphone to match their symmetry ratio with target. The therapists' team suggested a SpCV mode, in which direct muscle stimulation during swing phase contributed in enhancing the gait modification through afferent signal of vibration. In the current study, the proof-of-concept was demonstrated with a few subjects, but future studies with a larger population are needed to further evaluate and analyze these effects.

### 4.2. Study Limitations and Future Works

Following these positive initial results, the next step is to use this device in a study to determine long-term rehabilitation effects on subjects with gait abnormalities. In our current analysis the small number of participants is limitation of the data set; future research with a larger group of participants is obligatory. The implementation of rehabilitation devices in the daily life environment can help with progress in individuals with stroke. The proposed system is not limited to use with subjects suffering from stroke; it can also be applied to other patients suffering from gait asymmetry. Our current system features an inexpensive, portable, and easily operable device, the clinical functionality of which was demonstrated in this research. FSR-based insoles have 10–25 ms delay for gait phase detection mainly due to FSR properties. The vibrotactors used in our system have lag time of 40 ms and rise time of 87 ms. But these were oblivious to the participants and did not contribute in functionality of feedback provision. Currently, the system requires pretrial calibration of the FSRs for precise gait measurements. Furthermore, the system's biofeedback update is based on information from the previous gait cycle; velocity changes during the subject's walking are not considered. The current implementation does not incorporate a double-stance phase during feedback assignment, which will be addressed in a future study. Further modifications in the current system can result in a fully patient-operable device to assist the subject in rehabilitation. Perhaps, the system paired with smart phone (using a Bluetooth transceiver instead of XBee) can provide data logging and analysis, for daily life use of the device outside the rehabilitation clinic. These modifications will provide a cost-effective, complete, self-usable rehabilitation system, which will be a valuable tool for wearable and independent gait feedback.

## 5. Conclusion 

It is evident from this research that the portable system described is suitable for clinical applications relating to gait symmetry and functional improvements. The system is capable of assisting physical therapists in training individuals with stroke suffering from hemiparesis. The portability and effectiveness of the system are the key features demonstrated in these experiments. From trial results with healthy young subjects, it was concluded that temporal information provided in StMCV mode was more effective than proportional intensity information provided from SrMPV, due to the difficulty of identifying variation in vibration intensity during walking. The vibrotactile feedback influenced the improvements in symmetry of gait with negligible disturbance to the balance of individuals with stroke during walking. The results show the importance of biofeedback in helping the subjects to simulate target symmetry ratios, which points towards an important addition to rehabilitation procedures. With further recommended modifications, the system will provide assistance to therapists during gait rehabilitation sessions. This will help in reducing the therapy cost with increased patient effort in recovering the gait abnormalities.

## Figures and Tables

**Table 1:** Details of individuals with stroke.

Patient	Gender	Age	Height	Weight	Side of hemiplegia	Cause of stroke
1	Male	65	163 cm	57 kg	Left	Infarction
2	Male	67	165 cm	70 kg	Right	Infarction
3	Female	75	165 cm	65 kg	Left	Infarction
4	Male	52	166 cm	62 kg	Right	Infarction

**Table 2:** Trial modes for subjects.

Vibration mode	Subjects	
	Healthy young	Individual with stroke
Without vibration	(1) Normal walk	(1) Normal walk
	(2) Left-side handicapped walk	
	(3) Right-side handicapped walk	

StMCV mode	(4) Left-side handicapped walk	(2) Normal walk
	(5) Right-side handicapped walk	

SrMPV mode	(6) Left-side handicapped walk
	(7) Right-side handicapped walk

SpCV mode		(3) Normal Walk

**Table 3:** Results of healthy young subjects.

Exp. *R*	Left-side handicapped			Right-side handicapped		
	Without vibration	StMCV mode	SrMPV mode	Without vibration	StMCV mode	SrMPV mode
	0.5	0.5	0.5	0.5	0.5	0.5
S1	0.891 (±0.16)	0.604 (±0.07)	0.573 (±0.08)	0.605 (±0.20)	0.478 (±0.09)	0.472 (±0.11)
S2	0.745 (±0.19)	0.540 (±0.09)	0.617 (±0.05)	0.587 (±0.19)	0.503 (±0.10)	0.556 (±0.06)
S3	0.703 (±0.20)	0.539 (±0.11)	0.621 (±0.10)	0.592 (±0.16)	0.504 (±0.08)	0.560 (±0.09)
S4	0.718 (±0.17)	0.594 (±0.04)	0.475 (±0.11)	0.539 (±0.18)	0.469 (±0.11)	0.489 (±0.07)
S5	0.729 (±0.12)	0.543 (±0.10)	0.656 (±0.09)	0.594 (±0.13)	0.506 (±0.09)	0.585 (±0.10)

**Table 4:** Results of subjects suffering from stroke (symmetry ratio, ML tilt, and ML acceleration).

Index	Symmetry ratio (*R*)			ML Tilt-RMS (degree)			ML acceleration-RMS (m/s^2^)		
Patient	Without vibration	StMCV mode	SpCV mode	Without vibration	StMCV mode	SpCV mode	Without vibration	StMCV mode	SpCV mode
1 (left hemi)	1.236 (±0.05)	1.136 (±0.19)	1.132 (±0.17)	4.910 (±0.65)	4.516 (±0.29)	4.802 (±0.96)	0.329 (±0.12)	0.295 (±0.23)	0.317 (±0.26)
2 (right hemi)	1.334 (±0.01)	1.078 (±0.26)	1.102 (±0.19)	6.329 (±0.89)	6.199 (±0.83)	6.505 (±0.52)	0.525 (±0.21)	0.469 (±0.16)	0.498 (±0.17)
3 (left hemi)	1.152 (±0.06)	1.114 (±0.10)	1.026 (±0.24)	4.491 (±0.77)	4.616 (±0.92)	4.768 (±0.60)	0.391 (±0.17)	0.339 (±0.11)	0.348 (±0.19)
4 (right hemi)	1.148 (±0.07)	1.095 (±0.24)	1.104 (±0.15)	5.985 (±0.71)	5.896 (±0.64)	5.914 (±0.88)	0.50 (±0.09)	0.497 (±0.14)	0.511 (±0.20)

**Table 5:** Results of subjects suffering from stroke (right stance, left stance, and gait speed).

Mode	Without vibration			StMCV mode			SpCV mode		
Patient	Right stance (s)	Left stance (s)	Gait speed (m/s)	Right stance (s)	Left stance (s)	Gait speed (m/s)	Right stance (s)	Left stance (s)	Gait speed (m/s)
1 (left hemi)	0.981 (±0.02)	0.803 (±0.02)	0.48	0.977 (±0.02)	0.860 (±0.02)	0.45	0.957 (±0.02)	0.847 (±0.02)	0.46
2 (right hemi)	0.760 (±0.02)	1.013 (±0.01)	0.40	0.885 (±0.02)	0.958 (±0.03)	0.38	0.848 (±0.02)	0.945 (±0.01)	0.41
3 (left hemi)	0.998 (±0.01)	0.867 (±0.02)	0.46	0.993 (±0.02)	0.892 (±0.01)	0.48	0.912 (±0.02)	0.887 (±0.02)	0.49
4 (right hemi)	0.890 (±0.01)	1.021 (±0.01)	0.39	0.972 (±0.03)	1.059 (±0.02)	0.38	0.976 (±0.01)	1.076 (±0.01)	0.39

**Table 6:** Pearson correlation coefficient for balance analysis of individuals with stroke.

	ML tilt (RMS)		ML acceleration (RMS)	
	Without vibration versus StMCV mode	Without vibration versus SpCV mode	Without vibration versus StMCV mode	Without vibration versus SpCV mode
Pearson correlation coefficient	0.998	0.995	0.970	0.975
